# A High-Precision CMOS Temperature Sensor with Thermistor Linear Calibration in the (−5 °C, 120 °C) Temperature Range

**DOI:** 10.3390/s18072165

**Published:** 2018-07-05

**Authors:** Chua-Chin Wang, Zong-You Hou, Jhih-Cheng You

**Affiliations:** Department of Electrical Engineering, National Sun Yat-sen University, Kaohsiung 80424, Taiwan; blazesky@vlsi.ee.nsysu.edu.tw (Z.-Y.H.); freedom753@vlsi.ee.nsysu.edu.tw (J.-C.Y.)

**Keywords:** thermistor, temperature variation, sensitivity, linear calibration

## Abstract

A high-precision Complementary Metal-Oxide-Semiconductor (CMOS) temperature sensor for (−5 °C, 120 °C) temperature range is designed and analyzed in this investigation. The proposed design is featured with a temperature range selection circuit so that the thermistor linear circuit automatically switches to a corresponding calibration loop in light of the temperature range besides the analysis of the calibration method. It resolves the problem that the temperature range of a single thermistor temperature sensor is too small. Notably, the output of the proposed design also attains a high linearity. The measurement results in a thermal chamber justifying that the output voltage is 1.96 V to 4.15 V, the maximum linearity error ≤1.4%, and the worst temperature error ≤1.1 °C in the temperature range of −5 °C to 120 °C.

## 1. Introduction

In recent years, consumer electronic products are often equipped with many sensors to detect the surrounding environmental conditions, e.g., voltage, current, temperature, etc., to facilitate a rapid and appropriate response. Temperature is considered one of the most important environmental factors. For example, high temperature usually leads to increased power consumption and even system breakdown. Industrial sensors utilized in those temperature sensing scenarios are classified into four categories: infrared, thermocouple, resistance thermometer, and thermistor. The infrared spectrum of the measured product is distinct in the different temperatures. Thus, infrared thermometer measures the temperature in light of the infrared spectra [1,2]. Two dissimilar electrical conductors with different temperature coefficients compose a thermocouple, where an electrical junction is formed [3,4]. The thermocouple will generate a weak voltage when the one conductor of thermocouple is applied with a thermal gradient. When the temperature is measured from one conductor, the temperature of the other conductor is derived from the weak voltage according to the Seebeck effect [5]. The resistance thermometer has a linearity feature between the temperature and resistance [6,7]. Thus, the resistance of the resistance thermometer rises as the temperature rises such that the temperature is obtained from the resistance measurement. The sensing principle of the thermistor is very similar as that of the resistance thermometer. The major difference is that its material is mainly made of ceramic polymer. Therefore, the size is smaller and the reaction is faster. Table 1 summarizes features of the above four temperature detection methods.

Thermistor is often used for industrially cost-effective applications because it has ruggedness, high sensitivity, accuracy, and low cost. However, the thermistor sensor has a serious flaw, namely, a highly nonlinear resistance-temperature characteristic [8]. The nonlinear characteristic will dramatically increase calculation circuit’s area and cost. Many researchers have devoted efforts to resolve this problem, e.g., [9,10]. Sarkar et al. uses an inverting operational amplifier with a linear correction resistor to correct the thermistor [10]. However, it has serious temperature error at the lowest and highest temperatures. Bandyopadhyay et al. proposed a linearization scheme for thermistor-based sensing in biomedical applications [11]. This circuit, however, has large output error due to many resistors with resistance variations. Kumar et al. showed a block diagram of a neural network based method [12]. However, it is extremely inconvenient in a space-limited scenario due to the demand of high computing power.

## 2. Architecture of Proposed Temperature Sensor

**Figure 1:**
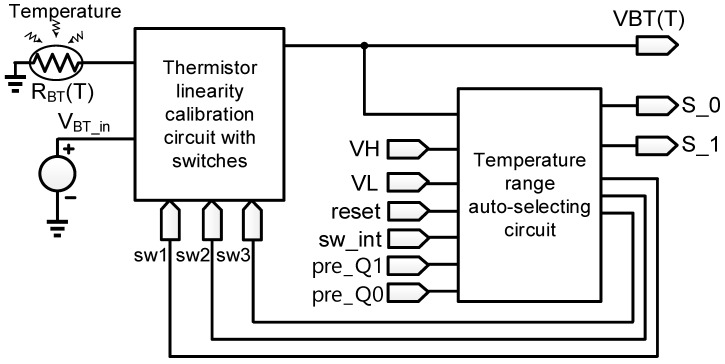
The block diagram of the proposed temperature sensor.

Figure 1 shows the proposed temperature sensor in this work, including a thermistor (R_BT_(T)), a thermistor linearity calibration circuit with switches, and a temperature range auto-selecting circuit. A thermistor linearity calibration circuit with switches is in charge of carrying out predictable linearization because the characteristic function of the thermistor is nonlinear. However, a single thermistor senses a relatively narrow temperature range. Besides, the linear correcting output, VBT(T), only attains high linearity when the sensed temperature is close to the selected center temperature. Therefore, the temperature range auto-selecting circuit selects feedback resistance and calibration resistance automatically to enlarge the sensing temperature range and enhance the linearity by detecting VBT(T). All of the sub-circuits in Figure 1 will be analyzed theoretically and designed in the following text. 

### 2.1. Thermistor Linearity Calibration Circuit with Switches

**Figure 2:**
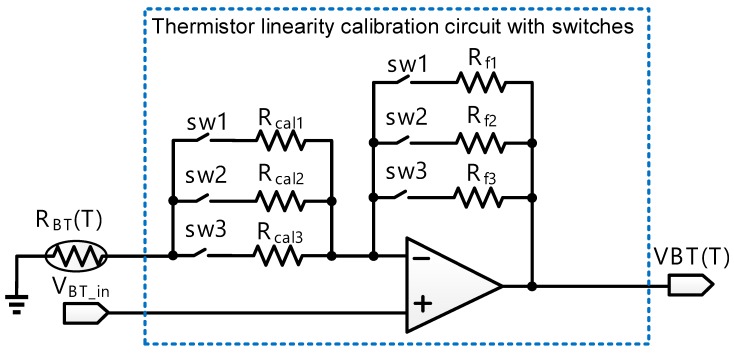
Schematic of thermistor linearity calibration circuit with switches.

**Figure 3:**
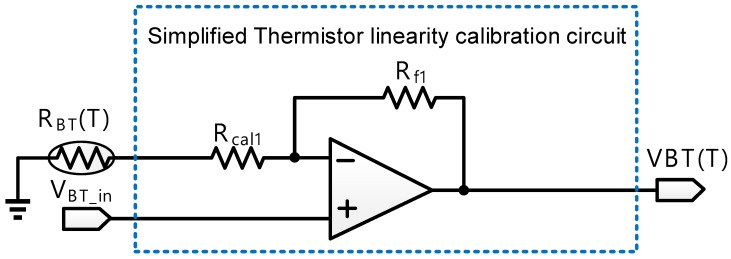
Schematic of simplified thermistor linearity calibration circuit. (sw1 on, and sw2 and sw3 off).

Referring to Figure 2, thermistor linearity calibration circuit with switches is composed of six switches, three calibration resistors, R_cal1_, R_cal2_, and R_cal3_, three feedback resistors, R_f1_, R_f2_, and R_f3_, and two operational amplifiers. Notably, V_BT_in_ is an external DC bias. Thermistor linearity calibration circuit with switches is used to enhance the linearity of the sensing voltage of the thermistor and reject the noise coupled from others. Take sw1 turn-on and sw2, sw3 turn-off as an example. Figure 2 then becomes Figure 3. The resistance of the thermistor is shown as follows:

(1) $$R_{\mathrm{BT}}\left(T\right){\;=\;R}_{0}e^{\beta (\frac{1}{T}\;-\;\frac{1}{T_{0}})}$$

where $R_{0}$ is the resistance of the thermistor at the 25 °C, β is the temperature constant of the thermistor, T is the sensed temperature, and $T_{0}$ is 25 °C. VBT(T) can be written as Equation (2):

(2) $$\mathrm{VBT}\left(T\right){\;=\;V}_{\mathrm{BT}\_\mathrm{in}}\;\times \;[1+\frac{R_{f1}}{R_{\mathrm{BT}}\left(T\right){\;+\;R}_{\mathrm{cal}1}}].$$

All the terms in Taylor series expansion of Equation (2) are truncated except the first three terms. VBT(T) is re-organized as follows:

(3) $$\mathrm{VBT}\left(T\right)\;=\;\mathrm{VBT}\left(T_{c}\right)\;+\;\left({T\;-T}_{c}\right){\mathrm{VBT}}^{\prime }\left(T_{c}\right)\;+\;\frac{{{(T\;-\;T}_{c})}^{2}}{2!}{\mathrm{VBT}}^{\prime \prime }\left(T_{c}\right)$$

where $T_{c}$ is the central temperature of the selected temperature range. The coefficient of the quadratic term needs to be zero to linearize VBT(T). Thus, the coefficient of the quadratic term is expressed as Equation (4):

(4) $$\frac{d^{2}}{{\mathrm{dt}}^{2}}\mathrm{VBT}\left(T\right){|}_{{T\;=\;T}_{c}}=\frac{d^{2}}{{\mathrm{dt}}^{2}}\left\{V_{\mathrm{BT}\_\mathrm{in}}\;\times \;\left[1+\frac{R_{f1}}{R_{\mathrm{BT}}\left(T_{c}\right){\;+\;R}_{\mathrm{cal}1}}\right]\right\}\;=\;0.$$

Equation (4) only needs to deal with the rightmost term where $V_{\mathrm{BT}\_\mathrm{in}}$ is a constant. The quadratic differential of Equation (4) will be written as Equation (5):

(5) $$\frac{d^{2}}{{\mathrm{dt}}^{2}}(1\;+\;\frac{R_{f1}}{R_{\mathrm{BT}}\left(T_{c}\right){+R}_{\mathrm{cal}1}}){|}_{{T\;=\;T}_{c}}\;=\;\frac{R_{f1}\left[R_{\mathrm{cal}1}{\;+\;R}_{\mathrm{BT}}\left(T_{c}\right)R_{\mathrm{BT}}^{\prime \prime }\left(T_{c}\right){\;-\;2R}_{\mathrm{BT}}^{\prime }{\left(T_{c}\right)}^{2}\right]}{{\left[R_{\mathrm{cal}1}{\;+\;R}_{\mathrm{BT}}\left(T_{c}\right)\right]}^{3}}{|}_{{T\;=\;T}_{c}}\;=\;0.$$

When Equation (5) holds, the numerator must be zero as follows:

(6) $$R_{\mathrm{cal}1}{\;+\;R}_{\mathrm{BT}}\left(T_{c}\right)R_{\mathrm{BT}}^{\prime \prime }\left(T_{c}\right){\;-\;2R}_{\mathrm{BT}}^{\prime }{\left(T_{c}\right)}^{2}=0.$$

$R_{\mathrm{cal}1}$ is then derived as follows:

(7) $$R_{\mathrm{cal}1}=\frac{{2R}_{\mathrm{BT}}^{\prime }{\left(T_{c}\right)}^{2}}{R_{\mathrm{BT}}^{\prime \prime }\left(T_{c}\right)}{\;-\;R}_{\mathrm{BT}}\left(T_{c}\right).$$

$R_{\mathrm{cal}1}$ is re-organized as Equation (8) according to Equation (1):

(8) $$R_{\mathrm{cal}1}\;=\;\frac{\beta \;-\;2\cdot T_{c}}{\beta \;+\;2\cdot T_{c}}\;{\times \;R}_{\mathrm{BT}}\left(T_{c}\right).$$

Namely, the coefficient of the quadratic term will become 0 by selecting $R_{\mathrm{cal}1}$ with an appropriate resistance. Besides, this work adds more calibration resistors and feedback resistors to enlarge the sensing temperature and maintain the high linearity of the VBT(T). Therefore, thermistor linearity calibration circuit with switches changes sensing temperature range by selecting the appropriate calibration resistor and feedback resistor. Referring to Equation (2), $R_{\mathrm{BT}}\left(T\right)$ is derived to attain the following result:

(9) $$R_{\mathrm{BT}}\left(T\right)=\frac{R_{f1}}{\left(\frac{\mathrm{VBT}\left(T\right)}{V_{\mathrm{BT}\_\mathrm{in}}}\right)-1}-R_{\mathrm{cal}1}.$$

According to Equations (2), (8), and (9), temperature (T) can be indirectly estimated by RBT(T) based on this conclusion:

(10) T=1(ln(Rf1(VBT(T)/VBT_in)−1 − Rcal1R0)β) + 1t0.

**Figure 4:**
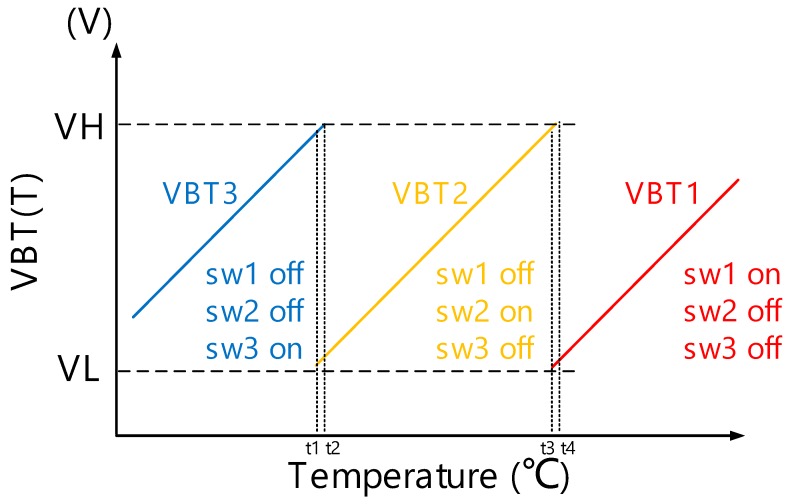
The illustration of VBT(T).

Figure 4 shows the illustration of the output of thermistor linearity calibration circuit with one of the three switches turned on. Namely, three different output voltage curves (VBT1, VBT2, and VBT3) are corresponding to the output voltage curves of VBT(T) in −5–35 ℃, 30–80 ℃, and 75–120 ℃, separately, in our design. VH (voltage high) and VL (voltage low), which are external DC bias voltages, are the reference voltages of temperature range auto-selecting circuit. VH is set to the maximum voltage of VBT2 and VBT3. By contrast, VL is set to the minimum voltage of VBT1 and VBT2. If VBT(T) > VH or VBT(T) < VL, temperature range auto-selecting circuit turn off the current switch and turn on another switch depending on VBT(T) higher than VH or lower than VL. On the other hand, when the VBT(T) is between VH and VL, it means that the thermistor linearity calibration circuit has been selected to the correct sensing temperature range. In addition, the sensing temperature ranges t1~t2 and t3~t4 are overlapping sensing temperature ranges of VBT3, VBT2, and VBT1, respectively. If there is no overlapping between two adjacent ranges, a bouncing hazard will occur between adjacent range selections when the temperature is close to the switch point of two adjacent ranges.

### 2.2. Temperature Range Auto-Selecting Circuit (TRASC)

**Figure 5:**
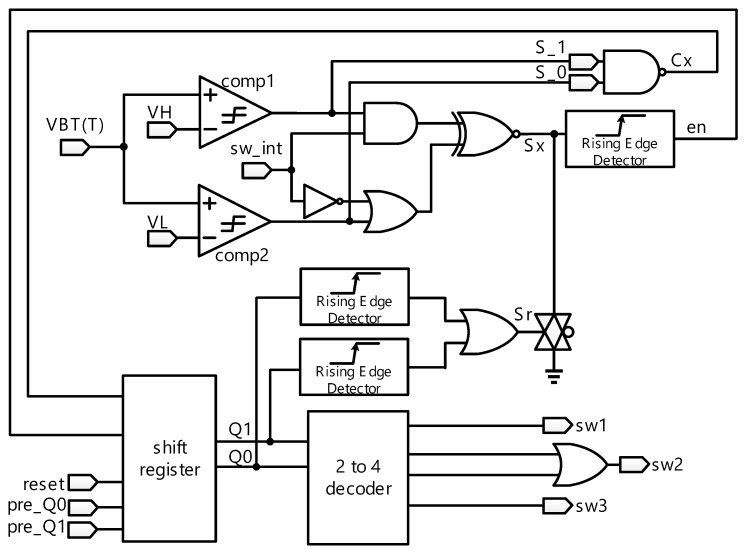
Schematic of temperature range auto-selecting circuit.

The temperature range auto-selecting circuit is shown in Figure 5, where two operating modes are used. The reset mode sets the initial state of temperature range auto-selecting circuit. The other is work mode, where VBT(T) is compared with VH and VL to auto-select corresponding resistors and enter the pre-defined sensing temperature range. The functionality of temperature range auto-selecting circuit will be described in detail in the following text. 

•  **Reset Mode**

When reset is low, Q1 and Q0 will be asserted low. By contrast, when reset is pulled up high and sw_int is dropped to low, Sx will be asserted low, sw1 and sw3 will be off, and sw2 will be on. Thus, VBT(T) follows the transfer function of VBT2. Finally, if sw_int is pulled up high, Temperature range auto-selecting circuit enters the work mode.

•  **Work Mode**

Temperature range auto-selecting circuit keeps tracking VBT(T) vs. VH and VL to determine which switch to turn on. If TRASC is kept in the work mode, Thermistor linearity calibration circuit with switches will be changed to appropriate temperature range according to the following rules.

◾ VBT(T) > VH: When VBT(T) is higher than VH, the sensing temperature region will change from VBT3 to VBT2 or VBT2 to VBT1 until VL < VBT(T) < VH. Notably, when VBT(T) is in the VBT1, the sensing temperature region stays the same.
◾ VL < VBT(T) < VH: When VBT(T) is between VH and VL, the state is kept in same temperature range.
◾ VBT(T) < VL: When VBT(T) is lower than VL, the sensing temperature region will change from VBT1 to VBT2 or VBT2 to VBT3 until VL < VBT(T) < VH. Notably, when VBT(T) is in the VBT3, the sensing temperature region stays the same.

**Figure 6:**
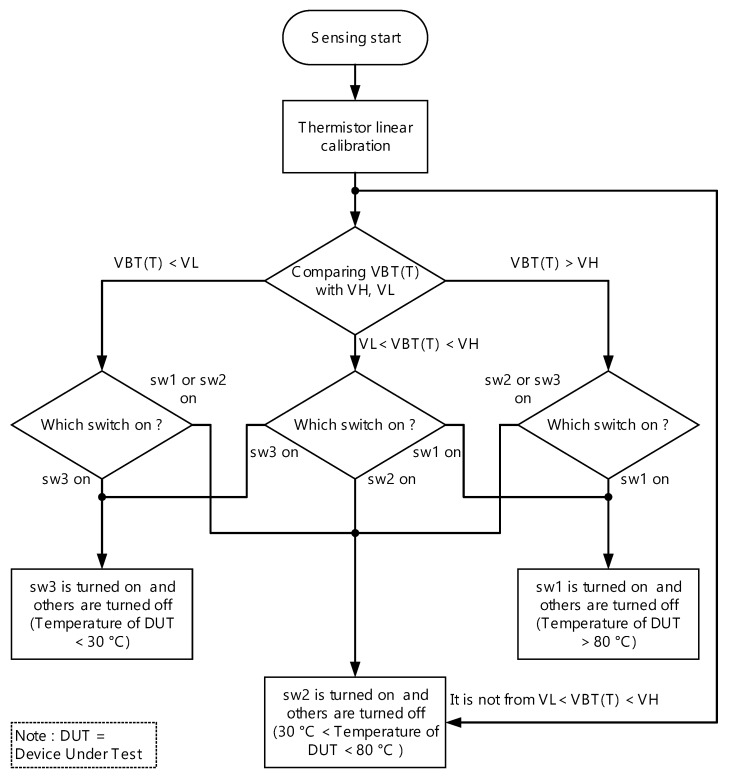
The flowchart of temperature range auto-selecting circuit (TRASC).

In short, Figure 6 shows the flow chart for TRASC how to select which switch be turned on.

**Figure 7:**
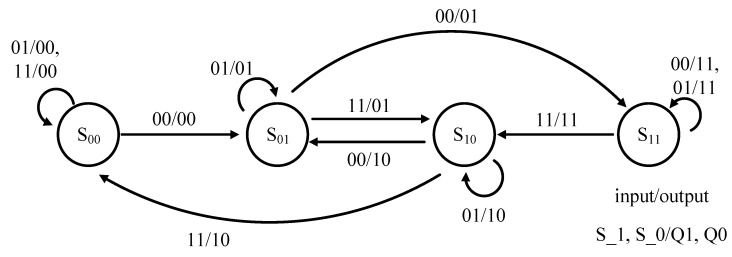
State machine of temperature range auto-selecting circuit.

A total of four states are required for temperature range auto-selecting circuit, which are S_00_, S_01_, S_10_, and S_11_. They are, respectively, described as shown in Figure 7. Referring to Figure 5 again, the inputs of this state machine is S_1 and S_0. By contrast, the outputs of this state machine is Q1 and Q0.

◾ S_00_ mode: sw1 is turned on and others are turned off. VBT(T) is in VBT1.
◾ S_01_ and S_10_ mode: sw2 is turned on and others are turned off. VBT(T) is in VBT2.
◾ S_11_ mode: The sw3 is turned on and others are turned off. VBT(T) is in VBT3.

## 3. Implementation and Measurement

**Figure 8:**
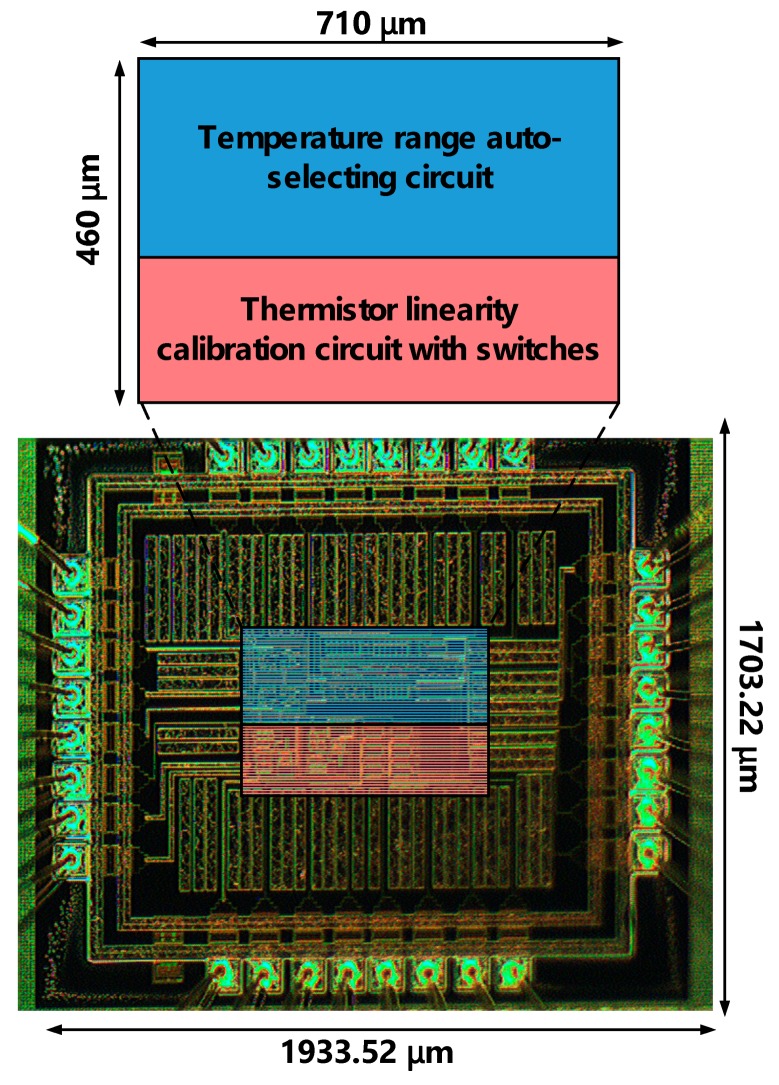
Die photo of the proposed temperature sensor.

**Figure 9:**
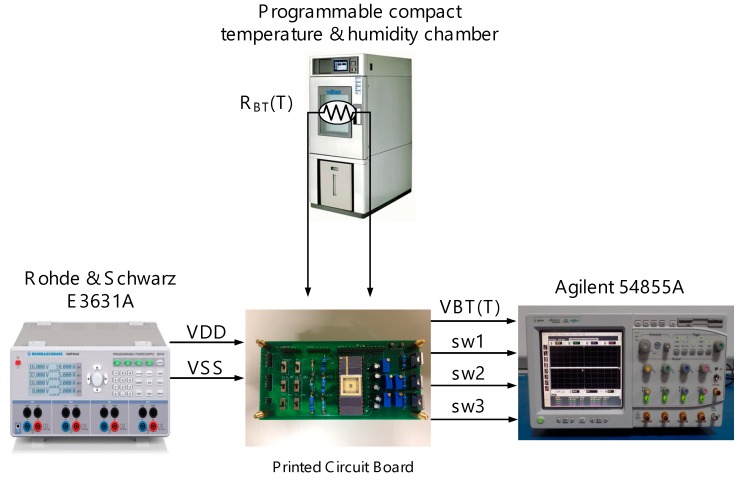
Measurement setup of the proposed Temperature sensor.

The proposed temperature sensor is carried out using TSMC 0.5 μm CMOS high voltage mixed-signal based LDMOS USGAL 2P3M polycide (T50UHV). Figure 8 shows the die photo of the proposed temperature sensor, where the entire chip area is 1933.52 μm × 1703.22 μm, and the core area is 710 μm × 460 μm. The measurement setup of the proposed design on silicon is shown in Figure 9. The thermistor is placed in the programmable compact temperature and humidity chamber (MHK-120 [13], TERCHY, Nantou, Taiwan). The power supply (E3631A DC Power Supply, Rohde and Schwarz, Munich, Germany) is in charge of the required supply voltages. The oscilloscope (Agilent 54855A) is used to demonstrate VBT(T).

**Figure 10:**
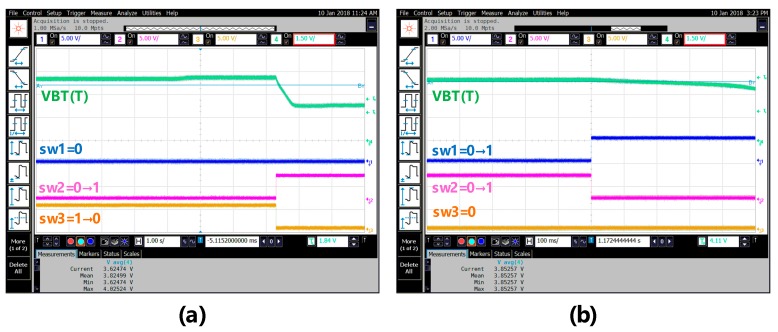
The scenario of three switches when the ambient temperature: (**a**) 35–40 °C and (**b**) 80–85 °C.

**Figure 11:**
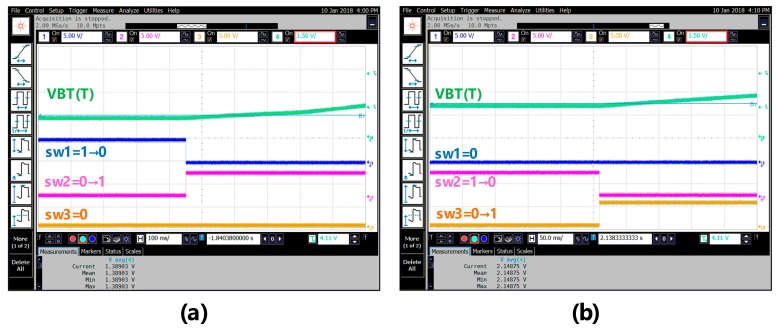
The scenario of three switches when the ambient temperature (**a**) 70–65 °C and (**b**) 30–25 °C.

**Figure 12:**
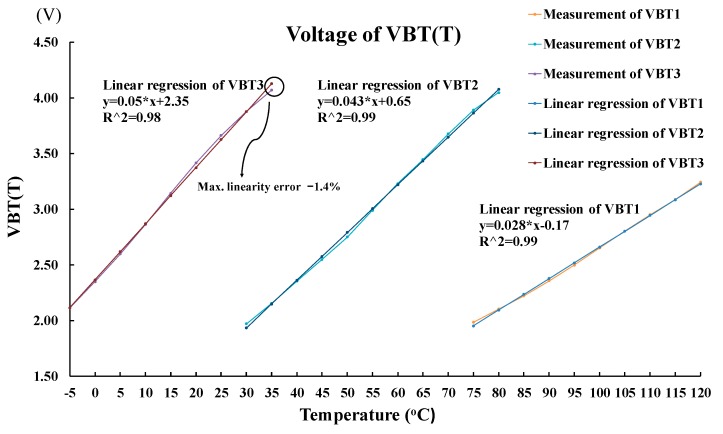
The measurement result of the VBT(T) with respect to a linear asymptotic line.

**Figure 13:**
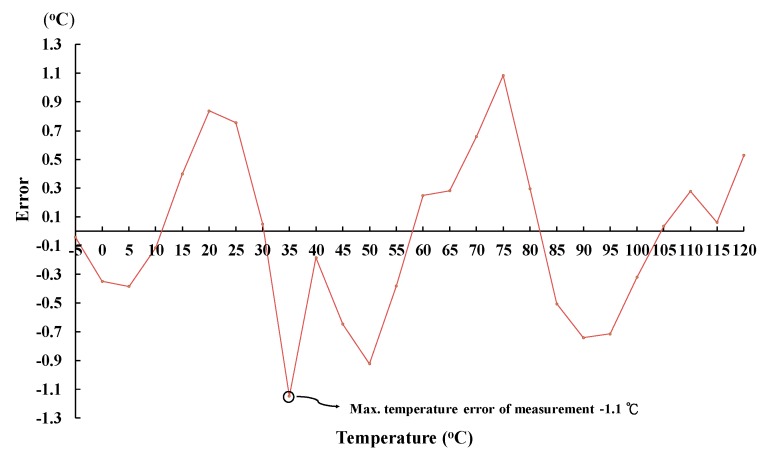
The measurement results of the proposed temperature sensor.

Figure 10 shows the scenario of three switches when the ambient temperature is changed: (a) from 35 °C up to 40 °C and (b) from 80 °C up to 85 °C. Referring to Figure 10, when the ambient temperature changes from low to high, the switch is turned on in sequence from sw3 to sw2 and then to sw1. By contrast, the scenario of the three switches, when the ambient temperature varies: (a) from 70 °C down to 65 °C and (b) from 30 °C down to 25 °C, is shown in Figure 11. Referring to Figure 11 again, when the ambient temperature changes from high to low, the switch is turned on in sequence from sw1 to sw2 and then to sw3. To make sure that the proposed chip is functional in the range of −5 °C to 120 °C, a total of 26 temperature cases, i.e., −5, 0, 5, …, 120 °C, are tested and measured. Figure 12 summarizes the measurement result of the VBT(T). The maximum error with respect to a linear asymptotic line is as low as 1.4%. Then, the error of the sensed temperature compared with the real temperature is attained in Figure 13. The maximum error with respect to real temperature is as low as 1.1 °C (≈3.1%).

**Figure 14:**
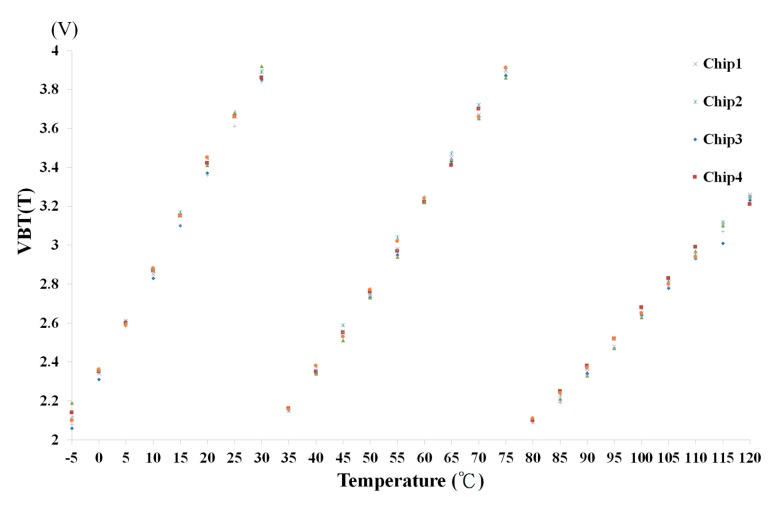
The summary of measurement results of the proposed temperature sensor.

**Figure 15:**
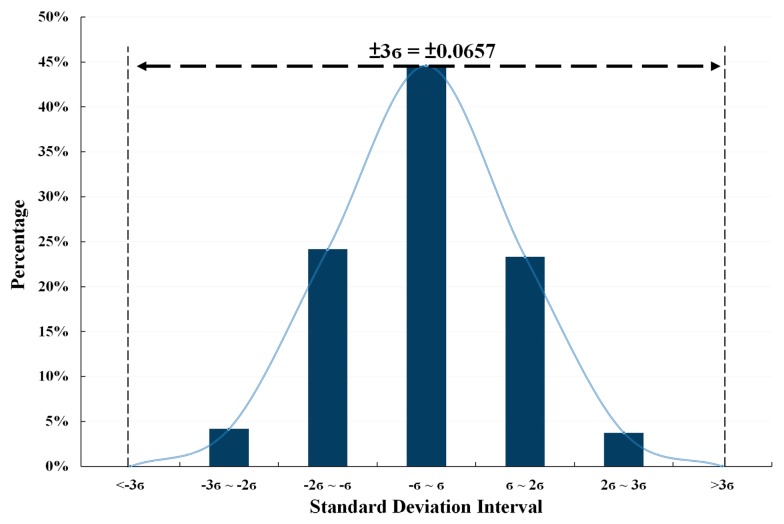
The standard deviation of the measurement results.

Four chips of the proposed temperature sensor are used to carry out the measurements, where each one was under five trials of 26 temperature tests. Figure 14 shows the summary of these measurement results. The standard deviation of measurement results is smaller than ±3σ as shown in Figure 15.

The performance comparison of the proposed design and several recent works is tabulated in Table 2. Notably, the proposed design attains the widest sensing temperature range, which is the range of −5 °C to 120 °C. Notably, the maximum error 1.1 °C, which is also the best to date.

## 4. Conclusions

In this paper, a temperature sensor with thermistor linear calibration with automatic temperature range selection is proposed. Detailed schematic design and analysis of the proposed design are given as well. The proposed design can extend sensing temperature range through a temperature range auto-selecting circuit. In addition, the measurement result of the proposed output demonstrates a high linearity, where the linearity error is smaller than 1.4%. Moreover, not only are the sensitivity and the sensing temperature range enhanced, the temperature error, which is less than 1.1 °C, is also better than that of the traditional temperature sensors by far.

## Figures and Tables

**Table 1:** The comparison table of the temperature sensor methods.

	Infrared	Thermocouple	Resistance Thermometer	Thermistor
Sensing temperature range (°C)	−50~400	−200~1200	−200~500	−90~150
Volume	≈200 × 50 × 50 mm	≈Ø20 × 2000 mm	≈Ø10 × 2000 mm	≈Ø0.5 × 1 mm
Reaction	Fast	Fast	Slow	Fast
Disadvantage	Limited measurement method	Need a reference temperature	Expensive	Nonlinear

**Table 2:** Performance comparison of temperature sensors.

	[14]	[10]	[11]	[15]	This Work
Year	2009 TIM	2013 SJ	2016 SJ	2017 HNICEM	2018
Implementation	PCB	PCB	PCB	PCB	T50UHV CMOS
Power (mW)	N/A	N/A	N/A	N/A	12
Output of Voltage (V)	N/A	2~4.5	N/A	N/A	1.9~4.1
Max. Linearity Error (%)	1.7	3	1.3	9.05	1.4
Sensing temperature range (°C)	0~120	30~120	30~110	−10~100	−5~120
Sensitivity (mV/°C)	N/A	27	N/A	N/A	17.6
Temperature Error (°C)	2	2.7	1.1	2	1.1
Reliability	N/A	N/A	N/A	N/A	≤±3σ
